# Work-life balance, job satisfaction, and burnout among nurses in Jordan: A cross-sectional study

**DOI:** 10.1371/journal.pone.0334603

**Published:** 2025-10-16

**Authors:** Sawsan Abuhammad, Karem H. Alzoubi, Sayer Al-Azzam, Reema Karasneh, Majed M. Masadeh, Mohamad Alameddine

**Affiliations:** 1 Department of Nursing, College of Health Sciences, University of Sharjah, United Arab Emirates; 2 Department of Maternal and Child Health, College of Nursing, Jordan University of Science and Technology, Irbid, Jordan; 3 Department of Pharmaceutical Sciences, College of Pharmacy, QU Health, Qatar University, Doha, Qatar; 4 Department of Clinical Pharmacy, Faculty of Pharmacy, Jordan University of Science and Technology, Irbid, Jordan; 5 Department of Clinical Pharmacy and Pharmacy Practice, Faculty of Pharmacy, Yarmouk University, Irbid, Jordan; 6 Department of Pharmaceutical Technology, Faculty of Pharmacy, Jordan University of Science and Technology, Irbid, Jordan; 7 Department of Health Care Management, College of Health Sciences, University of Sharjah, Sharjah, United Arab Emirates; Alhussain University College, IRAQ

## Abstract

**Aim:**

This study aims to investigate work life balance (WLB), job satisfaction, and occupational burnout among nursing professionals in Jordan. identifying key factors that influence their well-being and professional engagement.

**Methods:**

This cross-sectional study (January–April 2023) surveyed Jordanian nurses using the Netemeyer Work-Life Balance Scale, Job Satisfaction Scale, and Copenhagen Burnout Inventory. Stratified sampling ensured diverse representation. Multiple logistic regression analyzed WLB determinants, adjusting for demographics and work-related factors. Ethical approval was obtained from JUST-IRB, with informed consent and confidentiality assured.

**Results:**

A total of 500 nurses participated, 67.8% female. Key findings include nurses with a master’s or PhD degree reported significantly higher WLB than those with a bachelor’s degree (aOR = 3.081, p = 0.003). Work interference with personal life was evident, with 40.7% (165 respondents) reporting that their job demands negatively impacted their family life. In contrast, those working more than 50 hours per week had higher WLB (aOR = 2.652, p = 0.029).

**Conclusion:**

The study highlights the demographic and occupational factors influencing WLB, job satisfaction, and burnout among Jordanian nurses. Nurses working over 50 hours per week reported significantly higher WLB, yet job satisfaction remained moderate, and burnout, especially work-related, was a prevalent issue. Understanding these factors is crucial for enhancing the well-being and performance of nurses in Jordan.

## Introduction

Work-life balance (WLB) refers to an individual’s ability to effectively manage and integrate professional responsibilities with personal life, ensuring that neither aspect disproportionately interferes with the other. It is a dynamic equilibrium where work demands do not overwhelm personal time, relationships, or well-being, and vice versa [1].Literature reports a global surge in interest in understanding and systematically evaluating work-life balance (WLB). This is fueled by the significant impact that WLB has on job satisfaction, health outcomes, and professional performance [2,3]. Achieving a better WLB has been linked to increased organizational productivity, employee commitment, job satisfaction, and reduced absenteeism and turnover rates [4]. Factors contributing to WLB include insufficient work support, demanding work schedules, excessive workloads, and role conflicts [5,6].

The dynamic healthcare industry faces escalating and more complicated patient demands due to demographic shifts and epidemiological changes [7–9]. Adequate staffing with skilled professionals is crucial to meet these demands, yet, at many healthcare institutions, this is consistently counterbalanced by challenges such as high workloads, long work hours, staff shortages, and lack of flexibility. Those factors are exacerbating work-life imbalances among healthcare workers [10]. The aforementioned imbalances are exacerbated during public health emergencies and pandemics [11,12], particularly among nurses, who are usually disproportionally affected and who bear the highest-burden leaving them with little time for personal support and resilience-building activities [3,13]. The post-pandemic era has intensified workforce challenges in the nursing sector, with burnout and staff shortages becoming critical concerns. In Jordan, increasing healthcare demands, coupled with a high turnover rate among nurses, make work-life balance a pressing issue that requires immediate research and policy intervention. The imbalance between professional and personal life among nurses has severe consequences, including increased burnout, high turnover rates, and compromised patient care quality. The World Health Organization (2023) has identified nurse burnout as a significant threat to global healthcare sustainability, particularly in regions like the Middle East where nurse shortages are already critical.

Nurses in Jordan are held to rigorous clinical standards and are expected to maintain strict ethical practices while prioritizing the health and well-being of their patients [14]. Despite these clearly defined responsibilities, they often face the challenge of handling non-nursing tasks (NNTs) that detract from their primary caregiving roles [15]. These duties involve administrative responsibilities, clerical tasks, and other activities typically performed by different healthcare personnel [16]. Due to moral obligations and organizational demands, nurses are often compelled to take on roles outside their professional scope, which diverts their attention from essential patient care. Practical solutions include streamlining work environments to reduce NNTs by introducing efficient support services, which can help minimize neglected nursing duties and promote a healthier work-life balance [14].

The WLB reflects a nurse’s ability to juggle work, personal, and family demands [17,18]. Studies indicate that over 60% of nurses globally experience poor work-life balance, contributing to stress, burnout, and job dissatisfaction [19]. In Jordan, 72% of nurses report WLB struggles, with long working hours, high patient loads, and limited personal time cited as key issues [20]. Despite its critical role in nurse retention and healthcare quality, WLB among Jordanian nurses remains an under-researched area. Poor WLB can adversely affect nurses’ performance, leading to divided attention and decreased productivity [21]. Improving performance requires nurses to prioritize job satisfaction, which is enhanced by achieving set targets and aligning expectations with reality. Performance, influenced by the quality, quantity, and time dedicated to work, becomes especially critical as patient numbers rise [22]. This increased workload puts nurses under pressure as they try to balance their responsibilities at work and home, particularly for female nurses with families.

Job satisfaction, vital to nurse retention, is significantly influenced by work-life balance (WLB) [23]. Achieving WLB improves motivation, engagement, and commitment, fostering a sense of professional fulfillment and stability within the workforce [24]. Key factors influencing job satisfaction include fair pay, manageable workloads, strong organizational support, and opportunities for professional growth [25]. When nurses experience high job satisfaction, they demonstrate increased patient-centered care, lower stress levels, and greater overall performance [26]. Conversely, dissatisfaction may lead to workplace disengagement, higher absenteeism rates, and an increased likelihood of seeking employment elsewhere [27]. In Jordan, many nurses express dissatisfaction due to excessive workloads and a lack of career advancement opportunities, contributing to high turnover rates [24]. Implementing policies that support flexible work schedules, equitable workload distribution, and mental health resources can play a crucial role in enhancing job satisfaction and sustaining a stable healthcare workforce [25]. Institutions must also recognize the value of continuous professional development, leadership opportunities, and workplace recognition to ensure long-term job satisfaction among nurses.

Burnout, a direct consequence of poor WLB, leads to exhaustion, depersonalization, and reduced accomplishment [28]. High workloads, emotional strain from patient care, and inadequate organizational support contribute to burnout, making it a widespread occupational hazard in the healthcare sector [29] In Jordan, severe nursing shortages exacerbate the issue, forcing existing staff to work extended shifts with limited recovery time, further increasing the risk of burnout [30]. Burnout negatively impacts not only the well-being of nurses but also the overall quality of healthcare delivery. It has been linked to higher rates of medical errors, reduced patient satisfaction, and an increased probability of nurses leaving the profession altogether [28]. Addressing burnout requires a multi-faceted approach, including workload management strategies, structured mental health support programs, and professional development initiatives that help nurses build resilience against workplace stressors [31]. Hospitals and healthcare institutions must prioritize staff well-being by fostering supportive work environments, providing access to counseling services, and ensuring that nurses have adequate rest periods between shifts [32]. Without proactive measures to combat burnout, the Jordanian healthcare system risks further workforce depletion, resulting in greater patient care challenges and inefficiencies across medical institutions. Effective policies aimed at reducing burnout can lead to improved nurse retention, enhanced patient care outcomes, and a healthier work environment overall.

Achieving this balance requires workplace support. Consequently, there is a pressing need for interventions to safeguard and promote WLB among healthcare workers to protect their personal, professional, and psychological well-being. Although global studies have explored WLB in nursing [19,33], research in Middle Eastern contexts remains limited. A review by Alfuqaha et al [20] found that Jordanian nurses face significant WLB challenges due to long shifts and high emotional demands, yet few studies have examined the institutional and systemic factors influencing WLB in Jordanian healthcare settings. This study fills this gap by providing a comprehensive analysis of WLB among Jordanian nurses and its impact on job satisfaction and burnout. This study is essential in addressing the increasing burnout and turnover rates among Jordanian nurses by identifying key WLB challenges and proposing evidence-based strategies to improve retention. The findings can inform hospital administrators and policymakers in developing policies that support nurses’ well-being, ultimately enhancing patient care quality and healthcare system efficiency. This study aims to investigate WLB, job satisfaction, and occupational burnout among nursing professionals in Jordan.

## Methods

### Study design

The research employed a cross-sectional correlational approach.

### Theoretical framework

This research utilizes a theoretical framework that combines Maslach and Leiter’s [34] areas of the work-life model with Greenhaus et al.’s [35] theory of work-life balance. According to Maslach and Leiter [34], the alignment between an employee and their job across six areas of work-life significantly impacts their level of engagement or burnout. These areas include manageable workloads, autonomy, recognition, fairness, a sense of community, and values alignment between the individual and the organization. A ‘manageable workload’ refers to the extent to which physical and emotional job-related demands are sustainable given time and resource constraints. ‘Control’ represents the employee’s ability to make significant decisions about their work and access necessary resources. ‘Reward’ pertains to fulfilling an employee’s expectations through intrinsic and extrinsic recognition within the workplace. ‘Community’ reflects the quality of interpersonal relationships, including interactions with peers, supervisors, and subordinates. ‘Fairness’ relates to the perception of impartiality in decision-making processes and the support provided by management. Lastly, ‘values’ denote the alignment between the organization’s priorities and the employee’s ethical standards [36].

They found that excessive workload and perceived unfairness predicted emotional exhaustion, negatively impacting health outcomes. Greco et al. reported similar findings [37] among experienced acute care nurses. Other studies have linked person-job alignment in these work-life areas to turnover intentions mediated by burnout [38]. These findings align with the work-life model’s areas, highlighting how organizational factors influence employees’ work experiences but do not address personal factors, such as work-life conflict, which can also impact stress and job retention among new nurses.

### Instrument

The survey featured questions covering various sociodemographic aspects, including age, gender, marital status, educational attainment, occupation, employment sector, and years of professional experience.

### Work-life balance instrument

Work-life balance was assessed using the Netemeyer scale, a well-established instrument extensively validated by researchers [39,40]. This scale comprises ten items—five measuring work-family conflict and five assessing family-work conflict. A representative item from the work-family conflict section states, “My job responsibilities interfere with my home and family life,” while a sample statement for family-work conflict reads, “Stress from family obligations hinders my job performance.” The Netemeyer scale demonstrated high reliability, with Cronbach’s alpha values of 0.94 for work-family conflict and 0.93 for family-work conflict [39].

### Job satisfaction

The Job Satisfaction Scale (JSS), developed by Koeske et al. (1994), is a 14-item instrument designed to measure job satisfaction among human service professionals. The scale assesses three dimensions: intrinsic satisfaction, organizational satisfaction, and a two-item factor related to salary, promotion, and benefits. Across multiple studies involving over 600 helping professionals, the JSS demonstrated high internal consistency, with alpha reliabilities ranging from 0.83 to 0.91 for the full scale. The intrinsic and organizational satisfaction subscales showed reliabilities between 0.85 to 0.90 and 0.78 to 0.90, respectively

### Burnout inventory

The Copenhagen Burnout Inventory (CBI) measures burnout across personal, work-related, and client-related domains. Developed by Kristensen et al. [41], it focuses on exhaustion rather than depersonalization or reduced accomplishment, making it particularly useful in service and healthcare industries. The CBI has strong psychometric properties, with Cronbach’s alpha coefficients between 0.85 and 0.87, demonstrating high reliability. It also shows good construct validity, correlating well with other burnout measures, and has been validated across cultures. Research supports its role as a predictor of health outcomes, including absenteeism and stress-related illnesses [41].

### Recruitment process

This study employed cross-sectional study design among convenience sample of nurses in Jordan. Between January 5, 2023, and April 31, 2023, nurses working in healthcare settings received online invitations to participate in survey that using google forms. The inclusion criteria were nurses working in any health care settings and able to write and read in Arabic. This study exclude nurses on leave or those in non-patient-facing roles. The sample size for correlational design with a power level of 0.8, a significant alpha level of 0.05, and a moderate effect size of 0.25 based on G power was 450 participants. Adding a 15% attrition rate for 500 participants. The invitations directed participants to an online survey of google platform. Upon accessing the study website, participants were provided with detailed information and asked to consent to participate. Once consent was obtained, participants completed a baseline survey covering demographic and work-related information and topics related to WLB and staying healthy. The survey took approximately 15 minutes and was developed through a two-phase pilot process: (i) team members completed the survey and provided feedback for refinement; (ii) a sample of nurses who pilot-tested the updated survey to identify any remaining issues. Those nurses were excluded from studying. Pilot data were reviewed to ensure the integrity of the variables. Incomplete surveys were followed up with reminder emails, encouraging participants to update their contact details and complete the survey.

### Data processing and analysis

The Google Forms database was converted into SPSS datasets for cleaning and scoring. Missing data was handled systematically to ensure validity and reliability. Participation was voluntary, and valid response counts were reported with summary statistics. Logistic regression assumptions were assessed to ensure model validity. Multicollinearity was checked using Variance Inflation Factor (VIF) values, with no significant issues detected. The Box-Tidwell test confirmed the linearity of continuous predictors with the log-odds. Model fit was evaluated using the Hosmer-Lemeshow test (p > 0.05), Nagelkerke’s R², and the Likelihood Ratio Test, indicating a well-fitting model. Independence of observations was assumed, with robust standard errors applied where necessary. These checks confirmed the appropriateness of logistic regression for analyzing work-life balance, job satisfaction, and burnout among nurse. Logistic regression was conducted to determine the predictors for people with a high life-work balance. The assumptions associated with logistic regression encompass (a) adequate sample size, (b) absence of multicollinearity, (c) independence of residuals, and (d) identification and management of outliers. All analyses were conducted using SPSS with a significant level of 5%.

### Ethical approval

This study was reviewed and approved by the Institutional Review Board of the Jordan University of Science and Technology (JUST-IRB) with the approval number: [32/149/2022], dated [June-13–2022]. All participants provided electronic informed consent to participate in the study and for their data to be published. Confidentiality was achieved by not collecting any identifying data. Participants were assured that their participation was voluntary and would not affect their employment. All collected information was analyzed in aggregate format and stored on a password-protected computer with exclusive access to the research team.

## Results

Table 1 outlines the participants’ demographic and job-related details. Among the respondents, 67.8% (339) were females, and 32.2% (161) were males. The average age was 36.3 years, with a standard deviation of 6.4 years. Most participants were married, with 61.8% (309) reporting that their spouse was unemployed. Concerning children, 26% (130 respondents) reported having no children, 30% (150 respondents) reported having 1–2 children, and 44% (220 respondents) reported having three or more children. Regarding education, 60.6% (303 participants) held a diploma, 26% (130 participants) had a bachelor’s degree, and 13.4% (67 participants) held a master’s or PhD degree.

**Table 1:** Demographic and work-related characteristics of the study population (n = 500).

		n	%
*Demographic Characteristics*			
Age	(mean ± SD)	36.34	±6.42
Gender	Male	161	32.2
	Female	339	67.8
Marital status	Married – Spouse employed	148	29.6
	Married – Spouse unemployed	309	61.8
Number of children	0	130	26
	1-2	150	30
	3+	220	44
Highest degree	Bachelor of Nursing	130	26.0
	diploma	303	60.6
	Master’s or PhD	67	13.4
*Work-related Characteristics*			
Experience (years)	1-5	65	13
	6-10	120	24
	11+	315	63
Work Location	Hospital	268	53.6
	Others	232	46.4
Sector	Government public sector	435	87.0
	Private sector	65	13.0
Work Location	Amman	207	41.4
	Other cities	211	42.2
	Rural areas	82	16.4
Typical weekly working hours (including overtime)	1-39	153	30.6
	40-49	191	38.2
	50+	63	11.6
Overtime compensation	Not compensated	225	45
	Compensated	229	45.8
	Do not work overtime	75	15
Usual shift schedule	Morning/afternoon shifts	322	64.4
	Other shifts	178	36.4
Contractual entitlement to days off per week	Zero or 1 day	270	54
	2 days +	230	46.0
Number of nurses typically working during a shift	1 nurse	377	75.4
	2 nurses	77	15.4
	3 nurses +	44	8.8
Number of assistants typically working during a shift:	0 assistants	221	44.2
	1 technician	155	31.0
	2 assistants +	121	24.2

Participants’ job experience varied significantly, with 13% (65 respondents) reporting 1–5 years of experience, 24% (120 respondents) reporting 6–10 years of experience, and 63% (315 respondents) reporting 11 or more years of experience. Nearly half of the participants worked in hospitals (53.6%, 268 participants), while 46.4% (232 participants) worked in other settings. Most participants were employed in the government/public sector (87%, 435 participants), and 13% (65 participants) were in the private sector. Participants’ work locations were distributed among cities and rural areas: 41.4% (207 participants) worked in Amman, 42.2% (211 participants) in other cities, and 16.4% (82 participants) in rural areas. Regarding weekly working hours, 38.2% (191 participants) worked 40–49 hours per week, 30.6% (153 participants) worked 1–39 hours per week, and 11.6% (63 participants) worked 50 or more hours per week.

Regarding overtime compensation, 45.8% (229 participants) were compensated for overtime, 45% (225 participants) did not receive compensation, and 15% (75 participants) did not work overtime. Shift schedules varied, with 64.4% (322 participants) reporting working morning/afternoon shifts, while 36.4% (178 participants) worked other shifts (such as evening or night shifts). Additionally, 46% (230 participants) were contractually entitled to two or more days off per week, while 54% (270 participants) were entitled to one day off. The number of nurses per shift also varied, with 75.4% (377 participants) reporting one nurse, 15.4% (77 participants) reporting two nurses, and 24.2% (121 participants) reporting two or more nurses per shift. In comparison, 44.2% (221 participants) worked alone without any assistants.

### Work-life balance and job satisfaction

Table 2 provides a detailed summary of responses to the Work-Life Balance (WLB) Scale. The mean WLB score was 42.8 ± 9.9. Work interference with personal life was evident, with 40.7% (165 respondents) reporting that their job demands negatively impacted their family life. Additionally, 47.2% (191 respondents) mentioned struggling to meet family obligations due to the time required by their jobs. Furthermore, 42.7% (173 participants) noted that work prevented them from completing tasks at home, while 43.2% (175 respondents) felt that job-related stress hindered them from fulfilling family responsibilities. Over half (52.8%, 214 participants) admitted to altering family activity plans because of work.

**Table 2:** Description of the Work-Life Balance Scale (n = 500).

	Strongly disagree/ Disagree		Neutral		Strongly Agree/ Agree	
	n	%	n	%	n	%
1- The demands of my work interfere with my home and family life	141	34.8%	99	24.4%	165	40.7%
2- The time my job takes up makes it challenging to fulfill family responsibilities.	110	27.2%	104	25.7%	191	47.2%
3- Things I want to do at home do not get done because of the demands my job puts on me.	110	27.2%	122	30.1%	173	42.7%
4- My job produces strain, making it difficult to fulfil family duties.	96	23.7%	134	33.1%	175	43.2%
5- Due to work-related duties, I have to change my plans for family activities.	73	18.0%	118	29.1%	214	52.8%
6- The demands of my family or spouse/partner interfere with work-related activities.	109	26.9%	145	35.8%	151	37.3%
7- I have to put off doing things at work because of demands on my time at home.	138	34.1%	119	29.4%	148	36.5%
8- Things I want to do at work don’t get done because of the demands of my family or spouse/partner	171	42.2%	133	32.8%	101	24.9%
9- My home life interferes with my responsibilities at work, such as getting to work on time, accomplishing daily tasks, and working overtime.	147	36.3%	128	31.6%	130	32.1%
10- Family-related strain interferes with my ability to perform job-related duties.	155	38.3%	137	33.8%	113	27.9%

**Fig 1:**
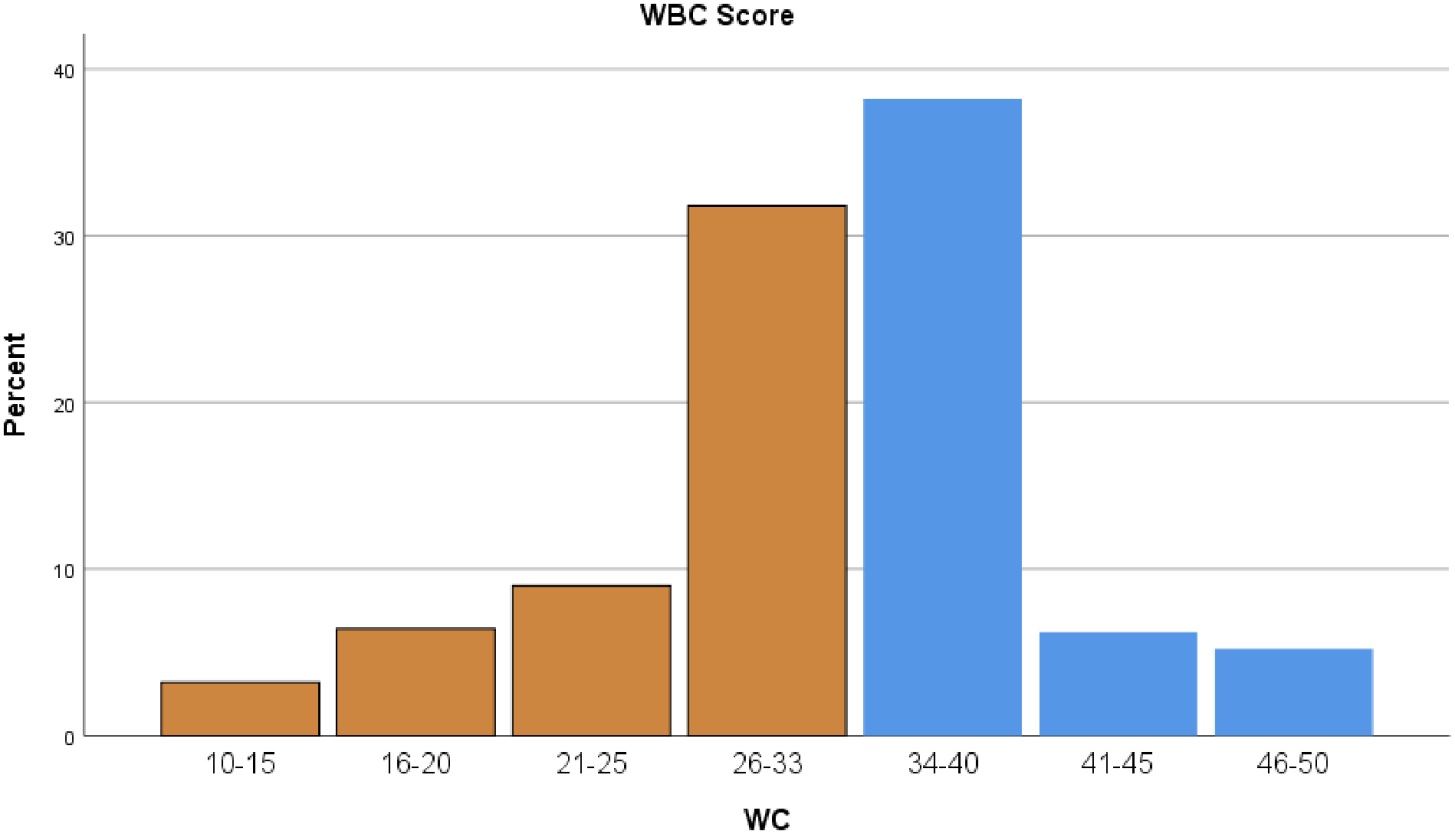
Distribution of work life balance scores for survey respondents M=42.8 + 9.9, Median=44, 50 percentile = 44, 75 percentile =50. Yellow for low balance and blue for high balance.

Conversely, 37.3% (151 respondents) acknowledged that family demands sometimes disrupt their work activities. In a related question, 36.5% (148 participants) reported postponing work tasks due to home responsibilities. In comparison, 32.1% (130 respondents) felt that personal life interfered with work duties, such as arriving on time or completing tasks. Opinions were divided on whether family-related stress impacted job performance, with 38.3% (155 participants) agreeing and 27.9% (113 respondents) disagreeing (Fig 1)

**Fig 2:**
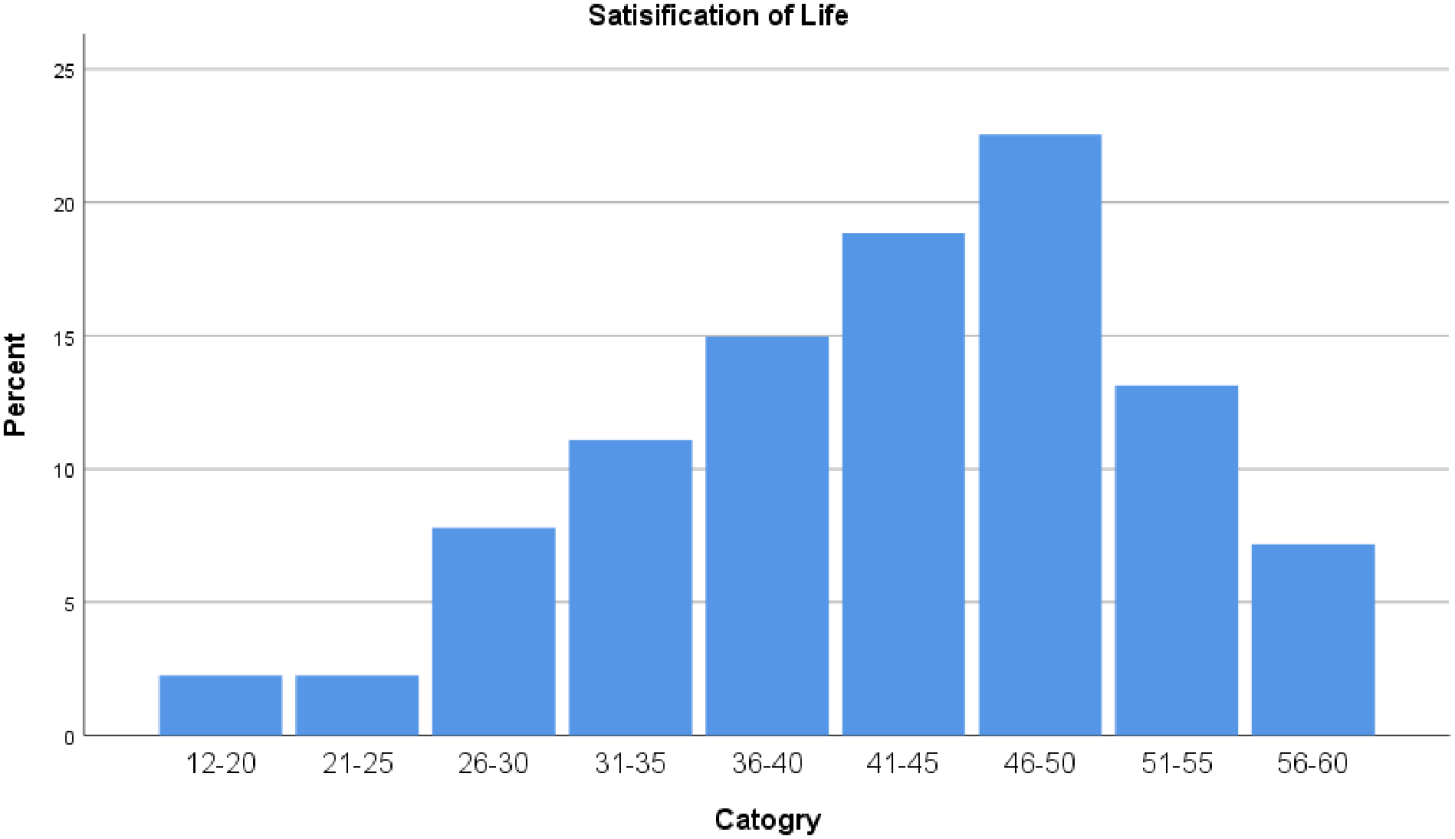
Distribution oj job satisfaction scores for respondents M = 32.6, Median = 33, 50 percentile = 33, 75 percentile = 39.

The survey participants had diverse opinions about their job satisfaction. The mean job satisfaction score was 32.6 ± 4.5. While 39.8% (161 respondents) expressed satisfaction with their jobs, 42.5% (172 respondents) were dissatisfied with the number of working hours per day. Furthermore, 43% (174 respondents) were content with their meal times and breaks, and 37.5% (152 respondents) were satisfied with their work environment. Many participants reported positive relationships with their managers (44.9%, 182 respondents) and co-workers (45.4%, 184 respondents), while 46.4% (188 respondents) found their work meaningful. However, 45.7% (185 respondents) were unhappy with their pay, and career growth opportunities were also a concern for 38.3% (155 participants). In terms of WLB, 37.8% (153 respondents) expressed dissatisfaction. Given these challenges, 31.6% (128 respondents) intended to change their nursing jobs within the next 12 months, and 22.7% (92 respondents) considered leaving the profession altogether. See Table 3 and Fig 2

**Table 3:** Description of the Job Satisfaction Scale (n = 500).

	Strongly disagree/ Disagree		Neutral		Strongly Agree/ Agree	
	n	%	n	%	n	%
1- I am satisfied with my job	137	33.8%	107	26.4%	161	39.8%
2- I am satisfied with the number of hours I work per day	172	42.5%	105	25.9%	128	31.6%
3- I am satisfied with how my shifts are scheduled	99	24.4%	162	40.0%	144	35.6%
4- I am satisfied with the time I have for breaks/meals	114	28.1%	117	28.9%	174	43.0%
5- I am satisfied with my salary	185	45.7%	125	30.9%	95	23.5%
6- I am satisfied with my work environment	104	25.7%	149	36.8%	152	37.5%
7- I am satisfied with the career growth opportunities I have	155	38.3%	149	36.8%	101	24.9%
8- I am satisfied with my personal life	129	31.9%	148	36.5%	128	31.6%
9- I am satisfied with my WLB	153	37.8%	140	34.6%	112	27.7%
10- I am satisfied with my relationship with my manager	87	21.5%	136	33.6%	182	44.9%
11- I am satisfied with my relationship with my co-workers	73	18.0%	148	36.5%	184	45.4%
12- I have the intention to change my job in the next 12 months within the pharmacy career field	148	36.5%	129	31.9%	128	31.6%
13- I have the intention to change my job in the next 12 months outside the pharmacy career field	160	39.5%	153	37.8%	92	22.7%
14- The work I do is meaningful	77	19.0%	140	34.6%	188	46.4%

### Occupational burnout

Table 4 highlights significant signs of burnout, particularly in the work-related domain. Most participants (46.7%, 189 respondents) reported moderate emotional exhaustion, and 33.6% (136 respondents) felt burnt out by their work. Client interactions were less exhausting, though 43.7% (177 respondents) felt they gave more to clients than they received. Additionally, 64% (259 respondents) frequently felt worn out by the end of the day, and 44.4% (180 respondents) reported morning exhaustion at the thought of another workday. About 33.3% (135 participants) found every working hour tiring, while 38.3% (155 respondents) still had enough energy for family and friends during their free time.

**Table 4:** Description of the Work-related Burnout Scale items (n = 500).

	To a low/ very low degree		Somewhat		To a high/ very high degree	
	n	%	n	%	n	%
1- Is your work emotionally exhausting? (WR)	55	13.6%	189	46.7%	161	39.8%
2- Do you feel burnt out because of your work? (WR)	127	31.4%	142	35.1%	136	33.6%
3- Does your work frustrate you? (WR)	123	30.4%	196	48.4%	86	21.2%
4- Do you find it hard to work with customers? (CR)	171	42.2%	144	35.6%	90	22.2%
5- Do you find it frustrating to work with customers? (CR)	151	37.3%	146	36.0%	108	26.7%
6- Does it drain your energy to work with customers? (CR)	145	35.8%	130	32.1%	130	32.1%
7- Do you feel you give more than you get back when working with customers? (CR)	89	22.0%	139	34.3%	177	43.7%
	**Never/ almost never/ Seldom**		**Sometimes**		**Often/ Always**	
8- Do you feel worn out at the end of the working day? (WR)	41	10.1%	105	25.9%	259	64.0%
9- Are you exhausted in the morning at the thought of another day at work? (WR)	68	16.8%	157	38.8%	180	44.4%
10- Do you feel that every working hour is tiring for you? (WR)	118	29.1%	152	37.5%	135	33.3%
11- Do you have enough energy for family and friends during leisure time? (WR)	127	31.4%	123	30.4%	155	38.3%
12- Are you tired of working with customers? (CR)	110	27.2%	152	37.5%	143	35.3%
13- Do you sometimes wonder how long you will be able to continue working with customers? (CR)	124	30.6%	147	36.3%	134	33.1%

### Predictors of WLB among nurses

The simple logistic regression analysis in Table 5 highlights several significant determinants of high WLB among nursing professionals. Nurses with a master’s or PhD degree have significantly higher WLB than those with a bachelor’s degree (aOR = 3.081, p = 0.003). In contrast, nurses with a diploma do not show a significant advantage (p = 0.193). Work-related characteristics reveal important insights: nurses with more than 50 working hours per week have a significantly higher WLB (aOR = 2.652, p = 0.029), while those working 40–49 hours per week do not exhibit a significant difference (p = 0.171). Additionally, those working evening or night shifts report higher WLB than those on morning/afternoon shifts (aOR = 1.726, p = 0.038). Nurses entitled to more than two days off per week demonstrate significantly higher WLB (p = 0.042). Lastly, burnout is a critical factor, with work-related burnout showing a strong negative association with WLB (aOR = 1.654, p = 0.014), while client-related burnout does not show a significant correlation (p = 0.855).

**Table 5:** Simple and Multiple Logistic Regression Analysis of Determinants for Work-Life Balance.

		Simple logistic regression				Multiple logistic regression			
		OR	p-value	95% CI		aOR	p-value	95% CI	
*Demographic Characteristics*									
Age	*(Continuous)*	0.980	0.155	0.953	1.008	0.976	0.316	0.930	1.024
Gender	Male	ref				ref			
	Female	0.836	0.355	0.572	1.222	1.051	0.857	0.610	1.811
Spouse employment status	Married – Spouse employed	ref				ref			
	Married – Spouse unemployed	0.171	0.449	0.805	1.705	1.509	0.175	0.833	2.734
	Single	0.618	0.156	0.89	1.9	0.638	0.440	0.204	1.997
Number of children	0	ref				ref			
	1-2	1.044	0.910	0.493	2.209	1.444	.360	.658	3.171
	3-4	0.953	0.865	0.544	1.668	.536	.524	.079	3.653
	5+	1.150	0.574	0.707	1.869	.602	.609	.087	4.193
Highest degree	Bachelor of Nursing	ref				ref			
	diploma	1.574	0.032	1.041	2.379	1.408	0.193	0.841	2.358
	Master’s or PhD	2.090	0.017	1.139	3.835	**3.081**	**0.003**	**1.469**	**6.463**
*Work-related Characteristics*									
Experience (years)	1-5	ref							
	6-10	0.737	0.328	0.400	1.359	0.756	0.482	0.346	1.650
	11+	0.833	0.511	0.484	1.435	1.212	0.654	0.523	2.808
Type of work	Hospital	ref				ref			
	Others	0.710	0.050	0.498	1.011	0.770	0.372	0.434	1.366
Sector	Government public sector	ref				ref			
	Private sector	1.074	0.791	0.634	1.817	0.766	0.365	0.431	1.363
Location	Amman	ref				ref			
	Other Cities	0.902	0.601	0.612	1.329	0.925	0.740	0.585	1.463
	Rural areas	0.614	0.063	0.367	1.027	0.856	0.638	0.446	1.640
Typical weekly working hours (including overtime)	1-39	ref				ref			
	40-49	1.647	0.010	1.124	2.414	1.436	0.171	0.855	2.413
	50+	2.027	0.029	1.075	3.822	**2.652**	**0.029**	**1.105**	**6.366**
Overtime compensation	Not compensated	ref							
	Paid	1.321	0.155	0.900	1.939	1.054	0.840	0.633	1.757
	Do not work overtime	1.517	0.125	0.890	2.586	1.028	0.930	0.549	1.925
Usual shift schedule	Morning/afternoon shifts	ref				ref			
	Evening/Night shifts	0.443	0.020	1.071	2.263	**1.726**	**0.038**	**1.031**	**2.891**
Contractual entitlement to days off per week	1 day	ref				ref			
	2 days +	1.065	0.738	0.738	1.536	0.607	0.042	0.375	0.983
Number of nurses typically working during a shift	1 nurse	ref				ref			
	2 nurses	0.662	0.047	0.440	0.995	1.162	0.607	0.657	2.055
	3 nurses +	0.627	0.060	0.386	1.020	1.336	0.278	0.792	2.254
Number of assistants typically working during a shift:	0 assistants	ref				ref			
	1 technician	1.403	0.310	0.730	2.695	.877	.686	.463	1.661
	2 assistants +	1.234	0.594	0.569	2.675	.510	.129	.214	1.217
Job Satisfaction (higher score = better job satisfaction)	*(Continuous)*	.965	.000	.947	.983	0.999	0.926	0.976	1.022
Burnout- Work related	*(Continuous)*	1.129	.000	1.074	1.186	1.654	0.014	1.106	2.475
Burnout – Client related	*(Continuous)*	1.142	.000	1.081	1.206	1.031	0.855	0.745	1.426

Abbreviations: OR: Odds Ratio, aOR: Adjusted Odds Ratio.

Values in bold are statistically significant.

## Discussion

This study is one of the first to investigate WLB among nurses in Jordan and underscores an imbalance between life and work among nurses. A concerning finding was that one-third of participants, particularly those in inpatient settings, felt unsupported by their employers in managing family responsibilities [42]. Nurses with dependent children reported greater difficulties in maintaining a satisfactory WLB compared to colleagues without children, aligning with findings that highlight the challenges of balancing parenting with work responsibilities [43]. Organizations should focus on providing adequate support and predictable work schedules for nurses with young children [44]. Addressing WLB is crucial, as research indicates that burnout is more likely to occur in individuals with poor WLB. Interestingly, some research suggests that nurses with children may experience less burnout, possibly because family responsibilities offer a form of relief from work-related stress [45]. This potential mediating effect warrants further investigation [46].

Our study found that nurses with a master’s or PhD have a significantly higher WLB than those with a bachelor’s degree. In contrast, nurses with a diploma do not have a significant advantage. This contributed to their reduced bedside responsibilities and greater seniority. This is consistent with international research, such as studies in oncology nursing, which have demonstrated that higher education levels often correlate with reduced burnout and improved satisfaction [47,48]. However, unlike previous findings, this study points to an educational disparity in WLB specifically among Jordanian nurses, underlining the need for local interventions to support nurses with lower educational qualifications.

Our study found that nurses working more than 50 hours per week reported significantly higher WLB, while those working 40–49 hours did not show a significant difference. Moreover, those working evening or night shifts reported a lower WLB than those on morning/afternoon shifts. Time off correlates with WLB, with nurses entitled to more than two days off per week demonstrating significantly higher WLB. Guveli et al. [47], which found that extended work hours were a significant contributor to stress and burnout in oncology nursing. This discrepancy suggests that financial incentives or differences in full-time versus part-time status might uniquely influence Jordanian nurses’ perceptions, requiring further investigation into these dynamics. This differences between our findings regarding working hours may be because of feeling more pressure and wanting to use their time wisely. Moreover, female-dominated professions like nursing, echoes findings in other contexts. For instance, studies in oncology nursing have shown similar challenges, where high work demands spill over into personal life, often leading to stress and burnout [42,45]. However, a study emphasizes a particularly acute experience for nurses in Jordan, especially those with family responsibilities. These findings suggest that while the work-life spillover issue is global, its intensity may vary based on cultural, systemic, and organizational factors.

Two studies confirm the strong inverse relationship between burnout and WLB [45,46]. Research in oncology nursing, such as the work of Gribben & Semple [48] has also highlighted this connection, with poor WLB being a key predictor of burnout. However, this study underscores that in Jordan, addressing WLB may be particularly effective in reducing burnout due to its central role in balancing professional and personal demands. Creating a supportive workplace culture that encourages learning, research, leadership development, and the identification of role models can improve nurses’ job satisfaction and reduce burnout [42,49,50].

### Implications for nursing education and nurse workforce

The present study found that many participants reported moderate emotional exhaustion and felt burned out by their work. Compared to high levels of burnout found in other studies [51,52]. This research highlights the potential to prevent burnout and recover from it by addressing personal and professional stressors. Proactive approaches must be taken to mitigate burnout before it becomes severe, as waiting for a formal diagnosis may have significant individual and organizational costs [53–55]. Healthcare institutions should take responsibility for identifying those at risk early on and providing training on stress management and resilience-building strategies [56,57]. This training is crucial since resilience has been identified as a critical trait for influential leaders in nursing and is a skill that can be developed to improve self-care [58]. Future research should investigate these unique findings further to refine strategies aimed at enhancing WLB and reducing burnout in Jordan and similar contexts.

### Limitations of the study

The study does not explore potential mediators such as financial incentives, personal resilience, or workplace support, which could help explain the counterintuitive results. This will be done for future studies. Future research could include mediation analyses to examine how financial incentives, personal resilience, and workplace support influence work-life balance in nursing. Collecting data through surveys or interviews could provide deeper insights into these factors, helping to design targeted interventions that enhance nurses’ well-being.

This was a cross-sectional study, which limits the generalizability of the findings. Longitudinal studies or qualitative investigations would provide valuable direction for further exploration of WLB in nursing. Moreover, the study does not discuss external factors such as economic pressures, hospital policies, or staffing shortages that may significantly impact WLB. Including an analysis of these stressors could offer a more comprehensive understanding of the challenges nurses face and inform practical recommendations for workplace improvements. Given that 67.8% of participants were female, it would be valuable to analyze gender and marital status as potential influencing factors in work-life balance. Future research might address this gap.

## Conclusion

This study provides a comprehensive analysis of work-life balance, job satisfaction, and burnout among nurses in Jordan. The results reveal that while some nurses report higher WLB despite long working hours, the overall workforce experiences moderate job satisfaction and significant burnout levels. Work-related burnout remains a critical issue affecting both personal well-being and professional performance. The study underscores the importance of workplace support in mitigating burnout and improving job satisfaction. Addressing these challenges is essential not only for the well-being of nurses but also for maintaining high-quality patient care and healthcare system efficiency.

## Supporting information

S1 Data Work-life balance. (XLSX)
